# Complete Genome Sequence of *Glaciecola psychrophila* Strain 170^T^

**DOI:** 10.1128/genomeA.00199-13

**Published:** 2013-05-02

**Authors:** Jinlong Yin, Jing Chen, Guiming Liu, Yong Yu, Lai Song, Xumin Wang, Xiaobo Qu

**Affiliations:** Changchun University of Chinese Medicine, Changchun, Chinaa;; CAS, Key Laboratory of Genome Sciences and Information, Beijing Institute of Genomics, Chinese Academy of Sciences, Beijing, Chinab;; SOA Key Laboratory for Polar Science, Polar Research Institute of China, Shanghai, Chinac;; Graduate University of Chinese Academy of Sciences, Beijing, Chinad

## Abstract

Here, we report the complete genome sequence of *Glaciecola psychrophila* strain 170^T^, a novel species of the genus *Glaciecola*, isolated from sea ice at high-latitude Arctic locations. The genome consists of a single chromosome (5,413,691 bp) and 5,363 genes. The genomics information will facilitate the study of the physiology, cold adaptation, and evolution of this genus.

## GENOME ANNOUNCEMENT

The genus *Glaciecola* was proposed by Bowman et al. (1) to accommodate Gram-negative, aerobic, psychrophilic, pigmented, and seawater-requiring bacteria. Members of the genus *Glaciecola* have been isolated from sea-ice samples collected from coastal areas of eastern Antarctica, marine invertebrate specimens, and polar seawater. Hitherto, the genus has comprised nine recognized species, including four psychrotolerant species, *Glaciecola polaris* (2), *Glaciecola chathamensis* (3), *Glaciecola nitratireducens* (4), and *Glaciecola agarilytica* (5); three psychrophile species, *Glaciecola punicea*, *Glaciecola pallidula* (1), and *Glaciecola psychrophila* (6); and *Glaciecola mesophila* (7) and *Glaciecola lipolytica* (8). Because of the wide temperature range tolerated for the growth of *Glaciecola*, it might be a good model to investigate bacterial cold adaptation mechanisms and evolution. *Glaciecola psychrophila* strain 170^T^ was collected from high-latitude Arctic locations (77°30′N to approximately 81°12′N), including the Canadian Basin and Greenland Sea. The temperature range for the growth of strain 170^T^ was 4 to 15°C, with optimum growth at 12°C, the lowest optimal temperature for a known species of the genus (6).

The *Glaciecola psychrophila* 170^T^ genome was sequenced with the 454 GS-FLX platform. A total of 158,121 high-quality reads with an average read length of 412 bp were produced, providing about 12-fold coverage of the genome. Assembly was performed using Newbler version 2.6, resulting in 142 large (defined as >500 bp) contigs. The relationship of contigs was determined by multiplex PCR, and gaps were filled through Sanger sequencing of PCR products by primer walking. The Phred/Phrap/Consed software package (9) was used for final sequence assembly and quality assessment. Protein-coding genes were predicted by combining the results of Glimmer 3.02 (10) and ZCURVE (11), followed by manual inspection. tRNA and rRNA genes were identified by tRNAscan-SE (12) and RNAmmer (13), respectively. Functional annotation was performed by searching against the NCBI nr, Swiss-Prot (14), InterProScan (15), COG (16), and KEGG (17) databases.

The single, circular chromosome constituting the genome of 170^T^ is composed of 5,413,691 bases, with a G+C content of 40.3%. It contains 5,636 open reading frames (ORFs), 6 rRNA operons, and 59 tRNA genes. There are 2,988 genes involving 23 COG function categories and 1,925 genes annotated into 1,799 KEGG orthology assignments by KAAS, involving 195 metabolic pathways. Furthermore, several genes potentially having a role related to cold adaptation were detected, such as genes for chaperonin GroEL, chaperonin GroES, RecA protein, and cold shock-like proteins. In summary, the sequenced genome of *Glaciecola psychrophila* 170^T^ not only facilitates research of the physiology, cold adaptation, and evolution of this genus but also opens up new opportunities to understand the functional genomics of this genus.

### Nucleotide sequence accession number.

The genome data have been deposited in GenBank under the accession number CP003837.
